# Quadriceps tendon autograft for primary ACL reconstruction: a Bayesian network meta-analysis

**DOI:** 10.1007/s00590-020-02680-9

**Published:** 2020-05-04

**Authors:** Filippo Migliorini, Jörg Eschweiler, Yasser El Mansy, Valentin Quack, Markus Tingart, Arne Driessen

**Affiliations:** 1 Department of Orthopaedics, University Hospital RWTH Aachen, Pauwelsstraße 30, 52074 Aachen, Germany; 2 Department of Orthopaedics, University Clinic of Alexandria, Alexandria, Egypt

**Keywords:** ACL reconstruction, Quadriceps, Hamstring, Patellar, Autograft

## Abstract

**Background:**

The purpose of the current study was to clarify the role of the quadriceps tendon (QT) autograft for primary ACL reconstruction. Thus, a Bayesian network meta-analysis comparing patients undergoing a primary ACL reconstruction with QT versus patellar tendon (PT) and hamstring tendon (HT) autografts was conducted.

**Material and methods:**

This Bayesian network meta-analysis was conducted according to the PRISMA extension statement for reporting systematic reviews incorporating network meta-analyses of health care interventions. In January 2020, the main databases were accessed. Articles comparing the outcomes of the QT autograft versus HT autograft and/or PT autograft for primary ACL reconstruction were included in the present study. The statistical analysis was performed with STATA Software/MP, through a Bayesian hierarchical random-effect model analysis.

**Results:**

Data from a total of 2603 knees were analysed. The overall mean follow-up was 35.0 months. Among the different grafts were evidenced comparable values of IKDC, Tegner and Lysholm score. The QT autograft detected comparable rate of Lachman test > 3 mm, Pivot shift test > 3 m and instrumental laxity > 3 mm. The QT autograft showed a lower rate of autograft failure above all. The QT autograft detected the reduced rate of AKP than the PT.

**Conclusion:**

Quadriceps tendon autograft may represent a feasible option for primary ACL reconstruction. These results must be interpret within the limitations of the present network meta-anlaysis.

## Introduction

The anterior cruciate ligament (ACL) stabilizes the knee and prevents anterior translation of the tibia in reference to the femur. According to epidemiological reports estimating 100,000 to 200,000 ACL ruptures per year in the USA alone, it is one of the most common injuries of active people [1]. If not repaired, a torn ACL can lead to joint instability, accelerate degenerative joint degradation and cause damage to meniscus and cartilage [2]. Therefore, surgical reconstruction in selected patients may be necessary [3]. Several surgical techniques such as allografts, autografts and synthetic grafts can be used for ACL reconstruction [4]. In patients with joint instability, the gold standard for ACL surgery is the reconstruction with an autologous tendon autograft [5]. Currently, the patellar tendon (PT) and hamstring (HT) autograft are believed to be the best available solution [6, 7]. Recently, there has been growing clinical interest regarding the role of the quadriceps tendon (QT) autograft for ACL reconstruction. Although this autograft has been used mainly in revision settings, some surgeons believe that this graft can achieve even better outcomes than standard PT and HT autografts also for primary ACL reconstruction. Clinical studies focusing on the use of the QT for primary ACL reconstruction report reliable long-term outcomes and low donor site morbidity rates [8]. Moreover, biomechanical proprieties of QT grafts promise to be the optimal for ACL reconstruction [9–11].

Currently, lack of evidence exists regarding the advantages of the QT as a source of autograft compared to PT and HT grafts. The purpose of the current study was to investigate the role of the QT for primary ACL reconstruction. A Bayesian network meta-analysis comparing patients undergoing a primary ACL reconstruction with QT versus PT or HT autograft was conducted.

## Material and methods

### Search strategy

This Bayesian network meta-analysis was conducted according to the PRISMA extension statement for reporting of systematic reviews incorporating network meta-analyses of health care interventions [12]. The preliminary protocol to guide the research was the following:

- P (Patients): primary ACL rupture;
- I (Intervention): QT autograft;
- C (Comparison): PT autograft, HT autograft;
- O (Outcomes): clinical scores, joint stability, anterior knee pain, failures.

### Literature search

Two independent authors (FM and AD) performed the data extraction. In January 2020, the following databases were accessed: Cochrane Systematic Reviews, Scopus, PubMed, EMBASE, CINAHL and Google Scholar. The following keywords were used for search either isolated or in combinations: resulting titles were screened by the authors. The full text of the articles of interest was accessed. The bibliography of the full-text articles was also screened.

### Eligibility criteria

Articles comparing the outcomes of the QT autograft versus HT and/or PT autograft for primary ACL reconstruction were included in the present study. According to the Oxford Centre of Evidence-based Medicine [13], level of evidence I to III were included. Articles in English, German, Italian, French and Spanish were considered. Cadaveric, biomechanics, computational and in vitro studies, as well as other meta-analysis or review studies, editorials, letters or expert opinions were excluded. Studies concerning QT autograft in revision settings were excluded. Only studies reporting data from a minimum 12-month follow-up were considered for inclusion. Only studies reporting quantitative data under the outcomes of interest were eligible. To reduce the indirect comparisons and to improve the evidence regarding the present Bayesian network meta-analysis, studies reporting data concerning HT versus PT autograft for primary ACL reconstruction were included. To be eligible for inclusion, high-quality papers were required, with sample randomization, comparability baseline, same techniques and protocols, and quantitative data under the same outcome of interest. Disagreements between the authors were debated and mutually solved.

### Outcomes of interest

Two independent authors performed data extraction (FM and AD). Studies generalities were extracted: author, year, type of study, level of evidence, number of patients and procedures, related mean age and duration of the follow-up. The outcomes of interest were: (1) clinical scores: subjective scale of the International Knee Documentation Committee (IKDC) [14], Lysholm Knee Scoring Scale [15], Tegner Activity Scale [16]; (2) joint laxity: Lachman test > 3 mm, Pivot shift test > 3 mm, arthrometer laxity > 3 mm; (3) post-operative complications: rate of failures and anterior knee pain (AKP). Concerning the instrumental laxity evaluation performed with an arthrometer, it was referred to as both KT-1000 and KT-2000 (MEDmetric Corp, San Diego, California). Both devices reproduce a linear force at 134 N to evaluate the knee laxity. These devices have been validated in other studies [17, 18]. A tibial displacement > 3 mm is considered as failure.

### Methodological quality assessment

For the methodological quality assessment, the Review Manager Software Version 5.3 (The Nordic Cochrane Centre, Copenhagen) was used. The risk of bias summary tool was performed according to the authors' judgements about each risk of bias item for each included study.

### Statistical analysis

The statistical analyses were performed by the senior author (FM). For the baseline comparability, the analysis of variance (ANOVA) test was performed through the IBM SPSS Software. Values of *P* > 0.5 were considered satisfactory. The statistical analysis was performed with STATA Software/MP, Version 14.1 (Stata Corporation, College Station, Texas, USA). The Bayesian hierarchical random-effect model analysis was adopted in all the comparisons. For continuous data, the generic inverse variance statistic method was adopted with the standardized mean difference effect measure (SMD). For binary data, the log odds ratio (LOR) effect measure was adopted. All the comparisons were matched with a reference value. Scores were compared with their maximum value, while dichotomic comparisons (rate of laxity and complications) with the null value. The edge network plot described contribution and nature of comparisons among studies. The interval plot was performed to rank the final effect of all the comparisons under the same endpoint. To evaluate the risk of publication bias, the funnel plot was performed in all the comparisons. The confidence interval (CI) and percentile interval (PrI) were set at 0.95 in all the comparisons. The overall inconsistency was evaluated through the equation for global linearity via the Wald test. If the *P* value > 0.5, the null hypothesis cannot be rejected, and the consistency assumption could be accepted at the overall level of each treatment.

## Results

### Search result

**Fig. 1:**
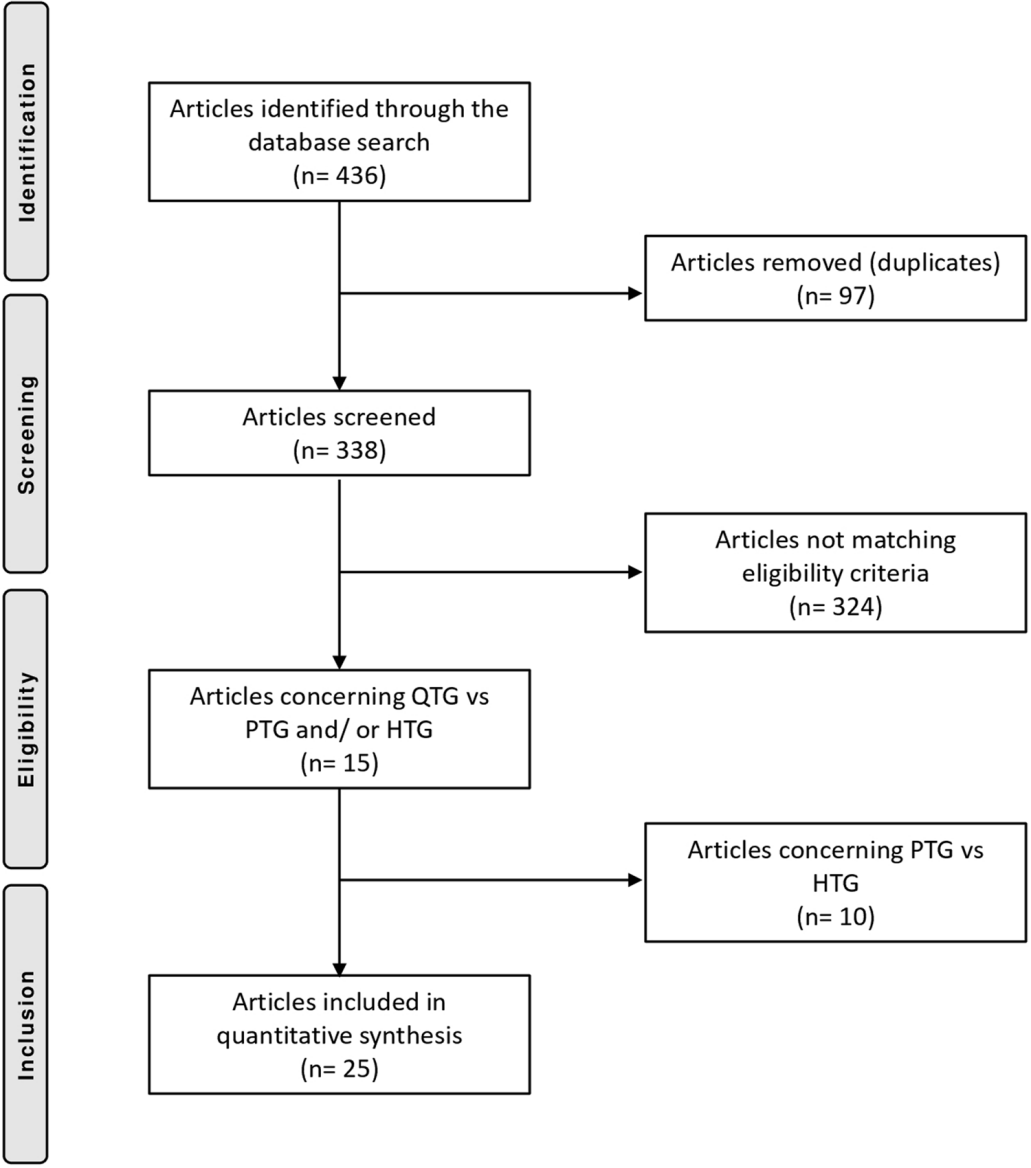
Flow chart of the literature search

Literature search and cross-references of the bibliography resulted in 436 studies. After excluding duplicates (97), further articles were excluded because they did not focus on the topic (141) or did not provide any comparison between the outcomes of interest (94), leaving 103 articles. After reading the abstracts, further 67 articles were rejected because incompatibility with the eligibility criteria was determined. During the full-text screening, 22 other articles were excluded because of a lack of quantitative data under the outcomes of interest. This left 15 papers treating QT versus PT or HT autografts. A total of 10 high-quality papers treating PT versus HT were added. This last operation left 25 articles for the present study. The flow chart of the literature search is shown in Fig. 1.

### Methodological quality assessment

**Fig. 2:**
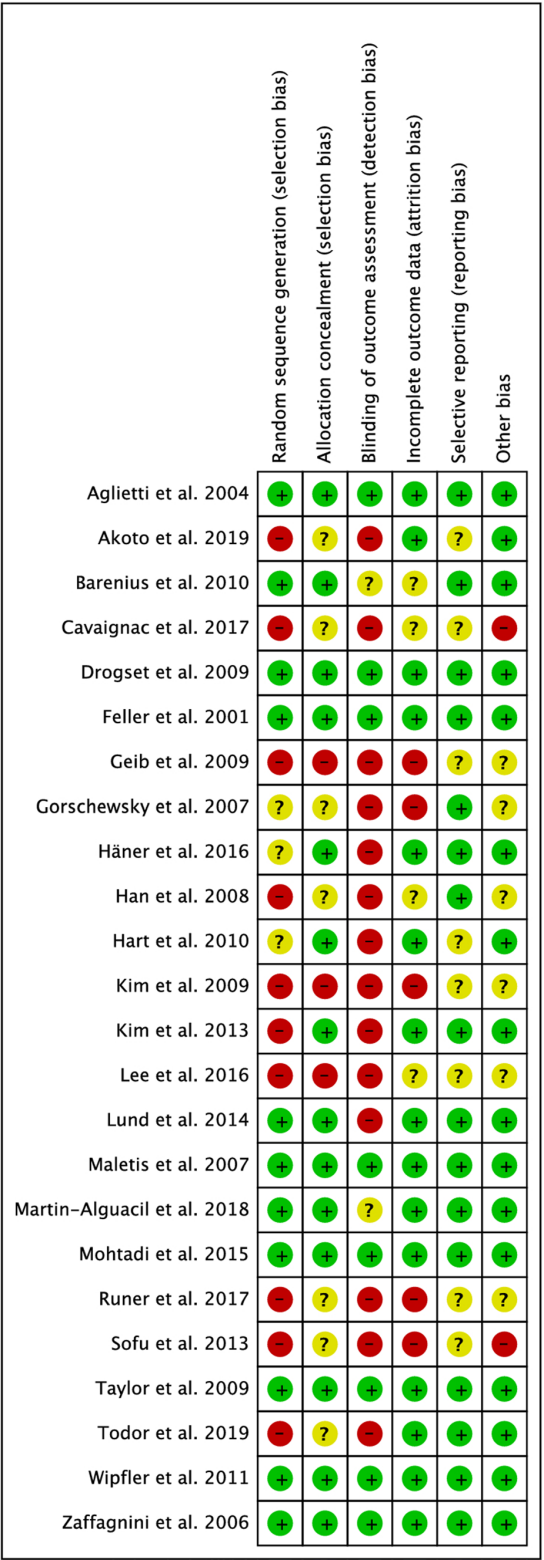
Risk of bias summary

The risk of bias summary detected a lack of blinding, especially among the QT autograft studies, improving the risk of detection bias. The randomization was promoted by 42% of the studies; therefore, the risk of selection bias was low in the 60% of the studies included. The risk of attrition and reporting bias was estimated as low in 65% of the studies included. The risk of unknown bias was estimated as low in 70% of the studies included. Therefore, a good quality of methodological assessment can be concluded for this work. The authors' judgement about each risk of bias item for each study included is shown in Fig. 2.

### Patient demographic

Data from a total of 2603 knees were analysed. The overall mean follow-up was 35.0 ± 24.8 months. In the QT group, a total of 856 knees were analysed. The mean age was 29.16 ± 1.6 years. In the HT and PT group, a total of 729 and 967 patients were analysed, respectively. The mean age in the HT and PT was 28.83 ± 3.4 and 28.60 ± 5.0, respectively. The ANOVA detected good comparability with regard to patients’ age (*P* = 0.8). Characteristics of the studies included and the demographic data are shown in Table 1.

**Table 1:** Characteristics of the included studies and demographic data

Author, year	Type of study	Procedures	Follow-up (months)	Type of autograft	Procedures	Mean age	Type of autograft	Procedures	Mean age
Aglietti et al. 2004 [19]	RCT	120	24.0	PT	60	25.0	HT	60	25.0
Akoto et al. 2019 [20]	RCS	82	12.0	QT	41	29.0	HT	41	28.0
Barenius et al. 2010 [21]	RCT	153	100.0	PT	78	33.0	HT	75	35.0
Cavaignac et al. 2017 [22]	RCS	86	43.2	QT	45	32.1	HT	41	30.1
Drogset et al. 2010 [23]	RCT	115	24.0	PT	58	26.0	HT	57	27.0
Feller et al. 2001 [24]	RCT	65	4.0	PT	31	26.0	HT	34	27.0
Geib et al. 2009 [25]	RCS	220	56.8	QT	190	31.7	PT	30	25.0
Gorschewsky et al. 2007 [26]	RCS	260	35.0	QT	124		PT	136	
Han et al. 2008 [27]	RCS	144	41.0	QT	72	27.8	PT	72	27.8
Häner et al. 2016 [28]	PCS	51	24.0	QT	25	35.9	HT	26	35.8
Hart et al. 2010 [29]	PCS	40	12.0	QT	20	27.0	HT	20	27.0
Kim et al. 2009 [30]	RCS	48	26.0	QT	21	27.1	PT	27	30.2
Kim et al. 2014 [31]	RCS	247	24.0	QT	89		PT	158	
		122	24.0	QT	53		PT	69	
Lee et al. 2016 [32]	RCS	96	35.0	QT	48	31.1	HT	48	29.9
Lund et al. 2014 [33]	RCT	51	24.0	QT	26	30.0	PT	25	31.0
Maletis et al. 2007 [34]	RCT	99	24.0	PT	46	27.2	HT	53	27.7
Martin et al. 2018 [35]	RCT	51	24.0	QT	26	18.7	HT	25	19.2
Mohtadi et al. 2015 [36]	RCT	206	39.0	PT	102	28.7	HT	104	28.5
Runer et al. 2018 [37]	RCS	80	24.0	QT	40	34.6	HT	40	34.6
Sofu et al. 2013 [38]	RCS	44	38.0	QT	23	26.8	HT	21	28.6
Taylor et al.2009 [39]	RCT	53	36.0	PT	24	21.7	HT	29	22.1
Todor et al. 2019 [40]	RCS	72	34.2	QT	39	30.6	HT	33	28.6
Wipfler et al. 2011 [41]	RCT	48	105.6	PT	26	40.0	HT	22	34.0
Zaffagnini et al. 2006 [42]	RCT	50	60.0	PT	25	31.0	HT	25	31.0

*RCS* retrospective cohort study; *PCS* prospective cohort study; *RCT* randomized clinical trial

### Results of the network comparisons: scores

**Fig. 3:**
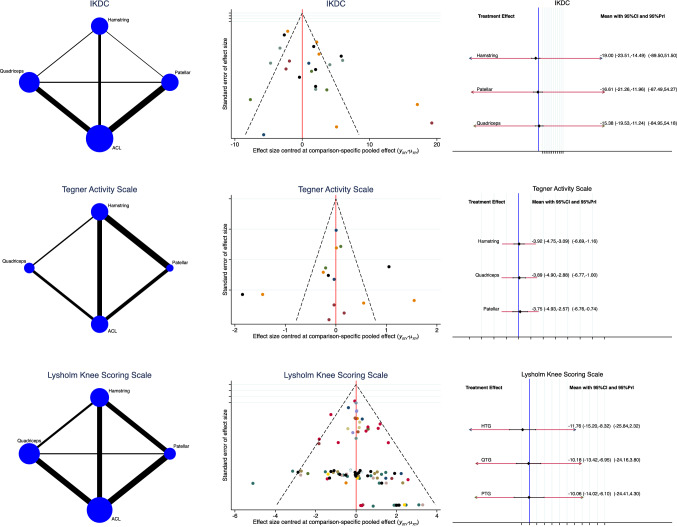
Overall network comparisons regarding the endpoint: scores

The QT scored worst in terms of IKDC (SMD:−15.38; 95% CI:−19.53,−11.24). Tegner (SMD:−3.89; 95% CI:−4.90,−2.88) and Lysholm scores (SMD:−10.18; 95% CI:−13.42,−6.95) were similar. The funnel plots resulted acceptable. The test for overall inconsistency found statistically significant similarity (*P* = 0.03, *P* = 0.09, *P* = 0.1, respectively). The analysis of these endpoints is shown in Fig. 3.

### Results of the network comparisons: laxity

**Fig. 4:**
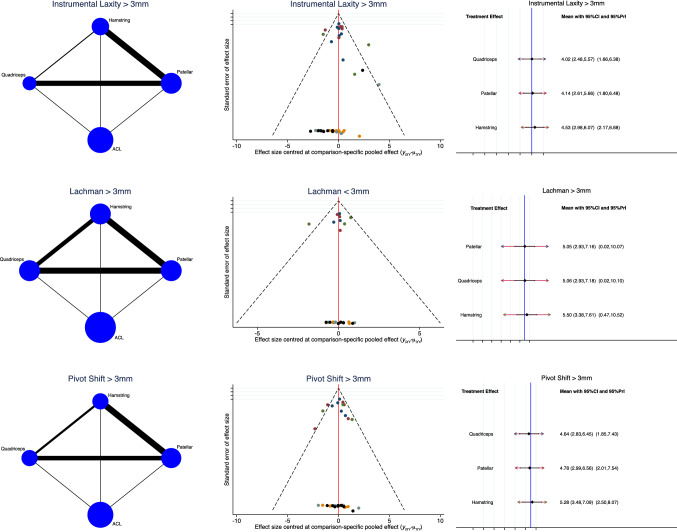
Overall network comparisons regarding the endpoint: laxity

Analysis of laxity resulted similar between the groups. The QT detected the reduced rate of instrumental laxity > 3 mm (LOR: 4.02; 95% CI: 2.48, 5.57) and intermediate values of Lachman (LOR: 5.06; 95% CI: 2.93, 7.18) and Pivot shift (LOR: 5.50; 95% CI: 3.38, 7.61) test > 3 mm. The funnel plots detected good and symmetrical distribution. The test for overall inconsistency found statistically significant similarity (*P* = 0.008, *P* = 0.02, *P* = 0.01, respectively). The analysis of these endpoints is shown in Fig. 4.

### Results of the network comparisons: complication

**Fig. 5:**
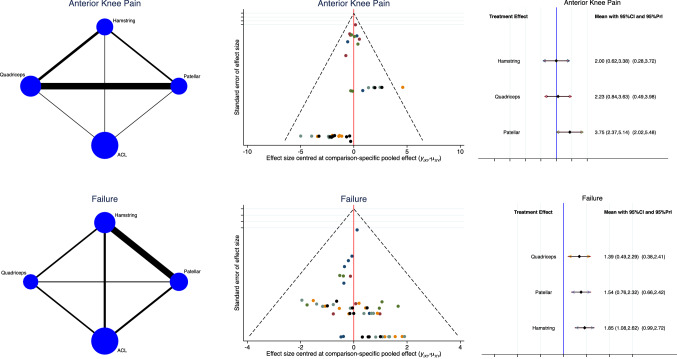
Overall network comparisons regarding the endpoint: complications

The results showed the lower rate of autograft failure (LOR: 1.39; 95% CI: 0.49, 2.29), followed by the PT (LOR: 1.54; 95% CI: 0.76, 2.32) and HT (LOR: 1.85; 95% CI: 1.08, 2.62). The rate of anterior knee pain for the QT (LOR: 2.23; 95% CI: 0.84, 3.63) was greater than that for the HT (LOR: 2.00; 95% CI: 0.62, 3.38) but lower than that for the PT (LOR: 3.75; 95% CI: 2.37, 5.14). The funnel plots were symmetric and very good distributed under the range of acceptability. The test for overall inconsistency through the equation for global linearity evidenced optimal transitivity in the network AKP and failures (*P* = 0.6, *P* = 0.6). The analysis of these endpoints is shown in Fig. 5.

## Discussion

The main findings of this Bayesian network meta-analysis are that the quadriceps tendon autograft may represent a feasible option for primary ACL reconstruction. The QT autograft reported lower rate of failure compared to both PT and HT autografts. Moreover, a reduced rate of AKP compared to the PT autograft has been observed. Analysis of joint laxity and clinical scores found comparability among the grafts.

The scores Lysholm and IKDC represent two valid and responsive patient-reported outcome measures (PROMs) to assess patients after ALC reconstruction [16, 43]. Tegner scale is one of the most frequent used activity rating score and is been validated also in studies on ACL reconstruction [16]. In the present network analysis, these scores detected similarity among the grafts. It is controversial and not fitfully clarified which autograft provides the best clinical outcome [23, 44, 45]. As evidenced by the funnel plots, low level of heterogeneity was observed. The assumptions through the equation for global linearity via the Wald test were refused, and the final result detected similarity between the autografts in terms of clinical scores of interests.

With regard to joint laxity, Lachman and Pivot shift tests were analysed as manual; the arthrometer KT-1000 and KT-2000 were used as an instrumental test to evaluate joint laxity. Reducing the articular laxity after an ACL rupture is one of the main objectives of surgery [46, 47]. In short term, joint laxity compromises the articular forces distribution, increasing the tendency to acute sprains and soft tissue lesions. Laxity can lead to articular degenerative changes such as osteoarthritis in the long term [48–50]. As evidenced by the funnel plots, low level of heterogeneity was observed. The assumptions through the equation for global linearity via the Wald test were refused, and the final result detected similarity between the autografts in terms of clinical scores of interests.

As far as stability is concerned, some biomechanical studies support the use of the QT autograft [11, 51]. Anatomical evidence states that QT is 50% thicker than the PT, providing an optimal stability of femoral condyles to the tibial plateau [52]. The PT autograft is considered the best choice in terms of its biomechanical properties and reduced revision rate [53]. However, the higher donor site morbidity (AKP) and the extension strength deficit led several surgeons to choose the HT autograft [54, 55]. Concern about the HT autograft is the flexion strength deficit in isokinetic testing [56–59]. Previous studies comparing the QT versus PT found a decreased incidence of donor site morbidity in the QT group [25, 33]. Several studies agree that HT autograft resulted in higher failure rate. Since the activation of the hamstring tendon reduces the load of the ACL providing knee stability [60, 61], its integrity may support the knee in valgus pivoting sport activities [62, 63], preventing autograft failure. In the present analysis, the use of QT grafts scored better in terms of both AKP with respect to the PT autograft and failure rate with respect to the HT autograft. These observations support the current use of QT for revision procedures but also improve future prospective as primary autograft.

The present study has some limitations. First, there are a limited number of eligible studies included. This is explained by the fact that the QT autograft procedure is still preliminary and not widespread. In support of this, our encouraging results should stimulate further studies, implementing the evidence of this procedure, the number of patients and the duration of follow-up. Moreover, the studies included provide a low level of evidence. In fact, there were eight retrospective cohort studies, impacting negatively to the overall evidence. Further studies should improve the scientific evidence, providing also a randomization method, thus reducing the risk of publication’s bias and the level of heterogeneity. The strength of the present study may be represented by the good baseline comparability and decent homogeneity of the results. Even though the level of evidence of this study is low, it provides a good set-up and analysis of the results.

## Conclusion

Quadriceps tendon autograft may represent a feasible option for primary ACL reconstruction. This autograft reported a lower rate of failure compared to both PT and HT grafts. Moreover, a reduced rate of anterior knee pain compared to the PT autograft has been observed. The analysis of joint laxity and clinical scores found comparability among the autografts. Orthopedic surgeon should consider these results in light of the evidence and limitations of this Bayesian network meta-analysis.
